# Phylogeography and phylogeny of Rhinoviruses collected from Severe Acute Respiratory Infection (SARI) cases over successive epidemic periods in Tunisia

**DOI:** 10.1371/journal.pone.0259859

**Published:** 2021-11-22

**Authors:** Sondes Haddad-Boubaker, Cherif Ben Hamda, Kais Ghedira, Khaoula Mefteh, Aida Bouafsoun, Ilhem Boutiba-Ben Boubaker, Amin Slim, Khaled Menif, Henda Triki, Mohamed Ali Ben Hadj Kacem, Hanen Smaoui

**Affiliations:** 1 Laboratory of Microbiology, Bechir Hamza Children’s Hospital, Bab-Saadoun Square, Tunis, Tunisia; 2 Laboratory of Bioinformatics, Biomathematics and Biostatistics, Institut Pasteur de Tunis, University of Tunis El-Manar, Tunis, Tunisia; 3 Microbiology of Children and Immunocompromised, Faculty of Medicine of Tunis, University of Tunis El-Manar, Tunis, Tunisia; 4 Laboratory of Microbiology, Charles Nicolle Hospital, Tunis, Tunisia; 5 Laboratory Research ‘‘Antimicrobial Resistance”, Faculty of Medicine of Tunis University of Tunis El-Manar, Tunis, Tunisia; 6 Pediatric Intensive Care Unit, Bechir Hamza Children’s Hospital in Tunis, Bab-Saadoun Square, Tunis Tunisia; Taif University, SAUDI ARABIA

## Abstract

Rhinoviruses (RV) are a major cause of Severe Acute Respiratory Infection (SARI) in children, with high genotypic diversity in different regions. However, RV type diversity remains unknown in several regions of the world. In this study, the genetic variability of the frequently circulating RV types in Northern Tunisia was investigated, using phylogenetic and phylogeographic analyses with a specific focus on the most frequent RV types: RV-A101 and RV-C45. This study concerned 13 RV types frequently circulating in Northern Tunisia. They were obtained from respiratory samples collected in 271 pediatric SARI cases, between September 2015 and November 2017. A total of 37 RV VP4-VP2 sequences, selected among a total of 49 generated sequences, was compared to 359 sequences from different regions of the world. Evolutionary analysis of RV-A101 and RV-C45 showed high genetic relationship between different Tunisian strains and Malaysian strains. RV-A101 and C45 progenitor viruses’ dates were estimated in 1981 and 1995, respectively. Since the early 2000s, the two types had a wide spread throughout the world. Phylogenetic analyses of other frequently circulating strains showed significant homology of Tunisian strains from the same epidemic period, in contrast with earlier strains. The genetic relatedness of RV-A101 and RV-C45 might result from an introduction of viruses from different clades followed by local dissemination rather than a local persistence of an endemic clades along seasons. International traffic may play a key role in the spread of RV-A101, RV-C45, and other RVs.

## Introduction

Rhinoviruses (RVs) are a common cause of upper respiratory diseases. In early childhood, they constitute a leading cause of severe lower respiratory infections causing important rates of hospitalization and mortality all around the world [1]. Clinical manifestations may vary from bronchiolitis and wheezing illnesses to pneumonia, and exacerbation of asthma [2, 3]. Besides, the infection may cause airway damage leading to subsequent asthma development [4].

RVs are non-enveloped, positive-sense RNA viruses, classified within the *Enterovirus* genus of the family *Picornaviridae* [5, 6]. They are characterized by a wide genetic diversity. Initially, they were classified into 101 distinct serotypes subdivided into two species, RV-A (76 serotypes) and RV-B (25 serotypes) [7]. Later, with the improvement of molecular-based diagnostic methods, new RVs were discovered [8] and RVs are now classified into three species, RV-A, RV-B, and RV-C, including 169 types [5]. In general, type identification is based on the VP4-VP2 coding region pairwise distances [9–11]. Nevertheless, using this approach, a significant number of RVs are still provisionally attributed. Thus, for accurate type identification, further genetic analysis using VP1 genomic coding region was also recommended [10, 11].

High genotypic diversity of RVs was reported in various regions of the world with co-circulation of different types during long periods or with turn-over of same types [12–14]. However, while RVs are highly variable viruses through mutations and recombination, leading to the emergence of divergent clades [15], studies on the genetic relationships between strains belonging to the same types remain scarce. It is not clear whether viruses from a RV clade can persist and establish continuous circulation and evolution in a geographic region or if RV circulation corresponds to the regular introduction of strains from different clades.

In Tunisia, we have recently described the circulation of 27 types of RV-A, B, and C, responsible for children’s SARI cases, between September 2015 and December 2017 [16]. Some of the circulating viruses were sporadic, while others circulated for long periods. In the present study, lineage variability and genetic relationships among the frequently circulating RV types in Tunisia were investigated for a better comprehension of RV molecular epidemiology and circulation dynamics. A particular concern was assigned to the most frequent types RV-A101 and RV-C45, using phylogeographic analyses.

## Material and methods

### Ethics statement

The study was approved by the local Ethical Committee of Bechir Hamza Children Hospital of Tunis, Tunisia. It was performed under ethical standards according to the 1964 Declaration of Helsinki and its later amendments. The sequences investigated in this study were obtained from samples collected for diagnostic purposes, as part of routine laboratory tests for a panel of respiratory viruses. The Ethical Committee waived the need for the consent of parents or guardians of the minors included in the study, since samples were obtained for diagnostic purposes and only viral sequences were investigated. The specimens were used after de-identification of the samples with respect to patient anonymity and patient data protection.

### Study population

Between September 2015 and November 2017, nasopharyngeal samples (n = 271) were collected from Tunisian children presenting Severe Acute Respiratory Infection (SARI) and admitted in the Pediatric Intensive Care Unit (PICU) of Bechir Hamza Children’s Hospital in Tunis. This hospital receives patients from Tunis (the capital), around 11 districts from northern Tunisia as well as complicated cases from other regions of the country. Among the 271 investigated samples, 57 were RV positive, using an in-house real-time PCR [16, 17]. Among the RV positive samples, 49 had sufficient residual samples to be used for VP4-VP2 PCR amplification and sequencing [7] and the 49 VP4-VP2 sequences were generated [16].

### RV VP4-VP2 sequences

The present study focused on the most frequently detected RV types in Tunisia, in the aim to evaluate the contribution of endemic circulation and virus importation from a region to another in the circulation dynamics of RVs. Thus, among the 49 generated sequences, 37 sequences, RV-A (n = 23); RV-B (n = 3) and RV-C (n = 11) were included. As we have previously demonstrated, they correspond to the most frequent RV types A and C in Northern Tunisian between September 2015 and November 2017: RV-A (8 types) and RV-C (3 types), and all RV-B circulating in the same period (3 types) (Table 1) [16]. Viral RNA was extracted using the QIAamp MinElute Virus Spin Kit (Qiagen, GmbH, Hilden, Germany) according to the manufacturer’s recommendations. The VP4-VP2 genomic region was amplified using the primer pair 9565-reverse and 9895-forward, producing a fragment of approximately 549 nucleotides [7]. The reaction was performed using the One-step RT-PCR kit from QIAGEN (GmbH, Hilden, Germany) in a total PCR mixture of 25μl containing 2.5μl of extracted RNA, 5μl of RT-PCR buffer, 400μM of each dNTP, 1μM of each primer, 1μl of enzyme Mix and RNase-free water. The thermal cycling conditions were as follows: 20mn at 50°C for reverse transcription, 15mn at 95°C for initial denaturation, 35 cycles of 1mn at 95°C for denaturation, 1mn at 60°C for annealing, 1mn at 72°C for the extension, and 7mn at 72°C for a final extension step.

**Table 1:** Description of Tunisian sequences investigated.

Sequence name	Date of isolation	Accession number	Type
HRV38.Tun.02.2016	Feb/2016	MN583163	RV-A101
HRV37.Tun.03.2016	Mar/2016	MN583162	
HRV04.Tun.03.2016	Mar/2016	MN583129	
HRV06.Tun.08.2016	Aug/2016	MN583131	
HRV25.Tun.09.2016	Sep/2016	MN583150	
HRV26.Tun.10.2016	Nov/2016	MN583151	
HRV29.Tun.01.2017	Jan/2017	MN583154	
HRV30.Tun.12.2016	Dec/2016	MN583155	RV-A32
HRV10.Tun.01.2017	Jan/2017	MN583135	
HRV14.Tun.04.2017	Apr/2017	MN583139	
HRV35.Tun.09.2015	Sep/2015	MN583160	RV-A12
HRV36.Tun.01.2017	Jan/2017	MN583161	
HRV11.Tun.02.2017	Feb/2017	MN583136	
HRV01.Tun.09.2015	Sep/2015	MN583126	RV-A15
HRV02.Tun.10.2015	Oct/2015	MN583127	
HRV03.Tun.11.2015	Nov/2015	MN583128	RV-A78
HRV45.Tun.10.2017	Oct/2017	MN583170	
HRV17.Tun.07.2017	Jul/2017	MN583142	RV-A16
HRV34.Tun.11.2017	Nov/2017	MN583159	
HRV20.Tun.02.2016	Feb/2016	MN583145	RV-A19
HRV24.Tun.08.2016	Aug/2016	MN583149	
HRV28.Tun.10.2015	Oct/2015	MN583153	RV-A89
HRV22.Tun.04.2016	Apr/2016	MN583147	
HRV13.Tun.03.2017	Mar/2017	MN583138	RV-C45
HRV42.Tun.03.2017	Mar/2017	MN583167	
HRV48.Tun.03.2017	Mar/2017	MN583173	
HRV16.Tun.05.2017	May/2017	MN583141	
HRV44.Tun.06.2017	Jun/2017	MN583169	
HRV39.Tun.06.2017	Jun/2017	MN583164	
HRV08.Tun.10.2016	Oct/2016	MN583133	RV-C43
HRV27.Tun.10.2016	Oct/2016	MN583152	
HRV46.Tun.10.2016	Oct/2016	MN583171	
HRV07.Tun.09.2016	Sep/2016	MN583132	RV-C53
HRV43.Tun.05.2017	May/2017	MN583168	
HRV32.Tun.08.2016	Jul/2016	MN583157	RV-B72
HRV31.Tun.12.2016	Sep/2016	MN583156	RV-B42
HRV49.Tun.03.2017	Mar/2017	MN583174	RV-B48

The VP4-VP2 amplicons were initially visualized in ethidium-bromide containing 1% agarose gel. The amplicons of the expected size were then purified by the Qiaquick PCR purification kit from Qiagen (Gmbh, Hilden, Germany). Sequences were obtained by automated sequencing using the Big Dye terminator chemistry, according to the manufacturer’s protocol (Applied Biosystems) in an automated sequencer (ABI 3130). Accession numbers of investigated sequences are indicated in Table 1.

### Data collection

In this study, a total of 359 VP4-VP2 RV sequences from several countries of the world, collected from the NCBI GenBank database (http://www.ncbi.nlm.nih.gov/nucleotide were investigated, in addition to the sequences obtained as part of the present study). Only well-annotated sequences, including the time of isolation and the geographic origin were selected. Duplicate sequences from same country same year were removed and a unique sequence of duplicates was retained. Phylogeographic analyses used 97 and 30 sequences of RV-A101 and RV-C45, respectively (S1 and S2 Tables). Phylogenetic analyses used 232 sequences of different RV types: RV-A12 (n = 27), RV-A78 (n = 24), RV-A32 (n = 21), RV-A89 (n = 14), RV-A15 (n = 21), RV-A16 (n = 11), RV-A19 (n = 24), RV-B72 (n = 22), RV-A48 (n = 13), RV-A42 (n = 15), RV-C53 (n = 18) and RV-C43 (n = 22). The accession numbers of the used sequences are indicated in the drawn trees.

### Bayesian phylogeography reconstruction

Bayesian analyses concerned the most circulating RV types in Tunisia, RV-A101 and RV-C45. Sequences were first aligned using Muscle 3.6 software [18]. Time-scaled phylogenies were inferred by Bayesian Markov Chain Monte Carlo (MCMC) sampling using BEAST v1.10.4 [19]. In a first step, the best substitution models were identified using the likelihood-based criteria (AIC) (S1 Fig), and the Smart Selection Tool (SMS) [20]. The time to the most recent common ancestor (tMRCA) was evaluated using the TN93 model with gamma invariant sites for RV-A101 and using the GTR model with gamma invariant sites for RV-C45 based on the SMS tool prediction. Five parametric models of coalescent population growth were then used: Bayesian skyline, constant population size, exponential growth, expansion growth and logistic growth under the strict, uncorrelated relaxed and random local clocks. The MCMC chains were run separately for each data set (RV-A101 and RV-C45). A total of 2 × 10^7^ MCMC iterations were run, with a burn-in of 2 × 10^6^ iterations and a sampling frequency of 6,000. In total, fifteen independent runs (five models under three clocks) were run. Convergence and effective sampling size (ESS) of estimates were assessed by visual inspection using Tracer v1.7 [21]. The best fitting models were then selected by the calculation of a Bayes Factor (BF) [22], using Marginal Likelihoods Estimation (MLE) mean of path sampling (PS) [23] and stepping-stone sampling (SS) (Tables 2 and 3) [24]. Finally, the Maximum Clade Credibility (MCC) trees with temporal and spatial annotation were constructed with a 10% burn-in removed using Tree Annotator being part of the BEAST package. Phylogenetic trees were generated using FigTree v1.4.2 (http://beast.bio.ed.ac.uk/figtree). Processed data sets were finally visualized using the “SPREAD 3” program on Google Earth Pro version 7.3 (http://www.google.com/earth/download/ge/) to produce a graphical animation of the continuous movement dynamics of RV-A101 and RV-C45 investigated sequences in the key markup language (kml) file format. These kml files contain information on the routes and times of virus movements, that can be displayed using Google Earth.

**Table 2:** Fitting models bayes factors and HPD95% related to RV-A101.

Clock/Tree prior	Constant	Exponential	Expansion	Bayesian	Logistic
	Size	Growth	Growth	Skyline	Growth
Random local	-2 048,50	-2 049,50	-2 048,52	-2 038,93	-1 513,96
	1980(1977,1982)	1980(1977,1982)	1980(1977,1982)	1980(1976,1982)	1981(1977,1982)
Strict	-2 048,11	-2 049,32	-2 049,10	-2 041,37	-1 555,08
	1980(1976,1982)	1980(1977,1982)	1980(1977,1982)	1980(1976,1982)	1980(1977,1982)
Uncorrelated Relaxed	-2 051,79	-2 055,36	-2 055,15	-2 042,35	-1 650,94
	1980(1977,1982)	1980(1977,1982)	1980(1977,1982)	1980(1977,1982)	1980(1977,1982)

**Table 3:** Fitting models bayes factors and HPD95% related to RV-C45.

Clock/Tree prior	Constant	Exponential	Expansion	Bayesian	Logistic
	Size	Growth	Growth	Skyline	Growth
Random local	-1 785,53	-1 788,06	-1 785,80	-1 784,33	-1 788,40
	1995(1989,1996)	1979(1961,1991)	1979(1961,1991)	1982(1942, 1996)	1982(1963,1999)
Strict	-1 792,07	-1 794,26	-1 794,06	-1 795,69	-1 791,73
	1943(1796,1977)	1947(1913,1975)	1944(1895,1971)	1950(1767, 1977)	1949(1928,1977)
Uncorrelated Relaxed	-1 781,67	-1 783,83	-1 781,15	-1 778,40	-1 783,60
	1993(1957,1996)	1993(1996,1987)	1993(1996,1987)	1995(1976, 1996)	1995(1992,1996)

### Phylogenetic analyses

In the aim to study genetic diversity and to identify the epidemiological origin of the other circulating RV types in Northern Tunisia, phylogenetic analyses were conducted using Mega version 6.0. (https://www.megasoftware.net/). The sequences were first aligned using Muscle 3.6 software. Dendrograms were drawn using the Neighbor-joining method and confirmed with 1000 bootstrap replicates.

## Results

### Epidemic history of RV-A101 and RV-C45

#### Evolutionary analysis

**Fig 1:**
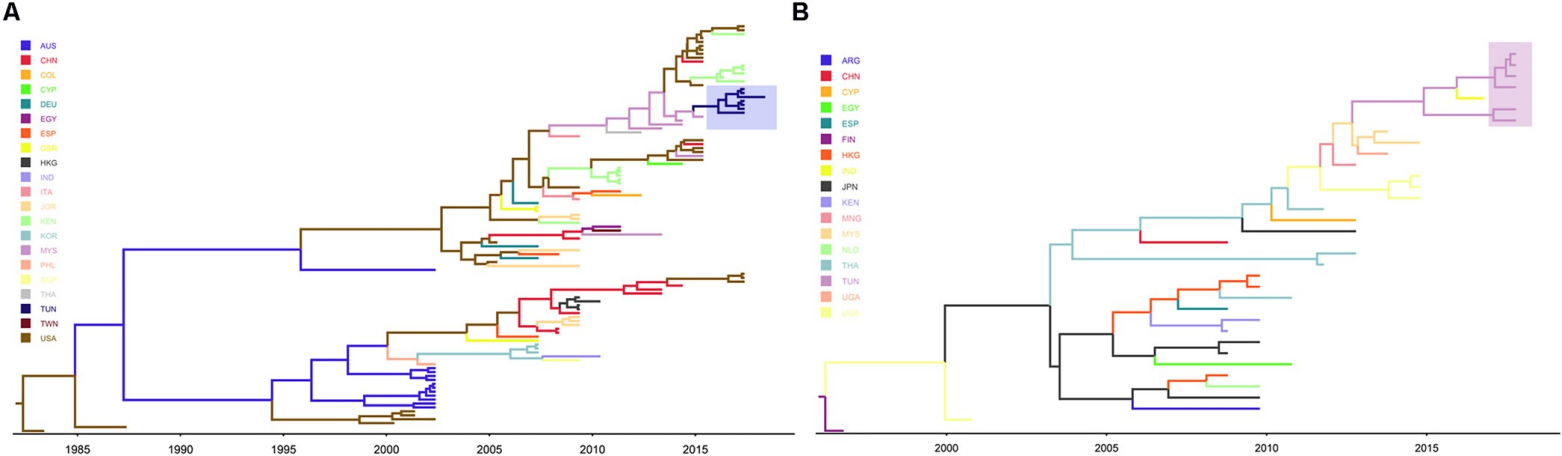
Maximum clade credibility (MCC) trees for RV-A101 (A) and RV-C45 (B). The branches are colored according to the most probable ancestor location of their descendant nodes. Tunisian RV-A101 and RV-C45 strains are highlighted in a Cyan and Purple rectangular, respectively.

Fig 1 displays the maximum clade credibility trees (MCC) of RV-A101 (Fig 1A) and RV-C45 (Fig 1B) for 100 and 36 sequences, respectively, from several countries. The tree in (Fig 1A) shows that at least four RV-A101 lineages are circulating in the world. However, no consistent geographical or time-related cluster could be identified. Tunisian sequences are highlighted with a cyan background color. The tree shows that almost all lineages are circulating in the USA. The Tunisian strains seem profoundly related to Malaysian ones. Likewise, several clades for RV-C45, could also be distinguished (Fig 1B) forming four potential monophyletic groups. The Tunisian sequences are highlighted with a purple background color. Tunisian strains seem extremely related to Mongolian, Malaysian, and Indian strains.

#### Bayesian skyline plots

**Fig 2:**
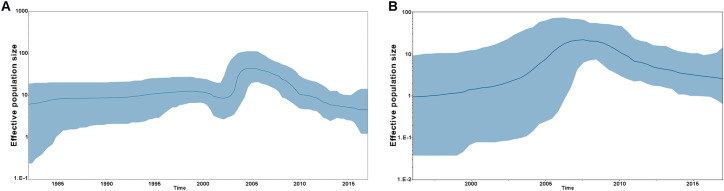
Bayesian skyline plot of RV-A101 (A) and RV-C45 (B). Bayesian skyline plots of the RV-A101 (A) and RV-C45 (B) genes show the changes in effective population size (genetic diversity) through time. The thick solid line indicates the median value, and the blue area is the 95% Highest Posterior Density (HPD) of the genetic diversity estimates.

We performed Bayesian coalescent analysis on the RV-A101 and RV-C45 sequences from Tunisia and other countries using five parametric models of coalescent population growth under the strict, uncorrelated relaxed, and random local clocks. For each demographic and molecular clock model, chain lengths of 20 million were used and sampled every 6,000 states. The data were analyzed using the Bayesian Skyline Plot (BSP) that represents the estimated change in the effective number of infected individuals over time for the RV-A101 and RV-C45 viruses. Fig 2 displays both Bayesian skyline plots corresponding to RV-A101 (Fig 2A) and RV-C45 (Fig 2B), respectively.

Fig 2A shows that the RV-A101 epidemic history was characterized by four phases of epidemic population growth including an initial period of relatively constant population size from 1980 to 2001, followed by a brief decline between 2001 and 2003, a third period of exponential growth until 2005 and a final phase with a significant drop from 2005 to 2019. Fig 2B shows that the RV-C45 epidemic history was characterized by two phases including an epidemic population growth period between 1980 and 2008 followed by a second period of a decline from 2008 to 2019. The waves on population size could be a consequence of an increase of international trades and exchanges during the last decades.

#### Most Recent Common Ancestor (tMRCA)

The progenitor virus of the RV-A101 that ultimately gave rise to all RV-A101 sequences was estimated around 1981 with an HPD (Highest Posterior Density) 95% [1977, 1982] and the one that eventually gave rise to all RV-C45 circulating in the world was estimated around 1995 with an HPD95% [1976, 1996], respectively.

#### Phylogeographic analysis

**Fig 3:**
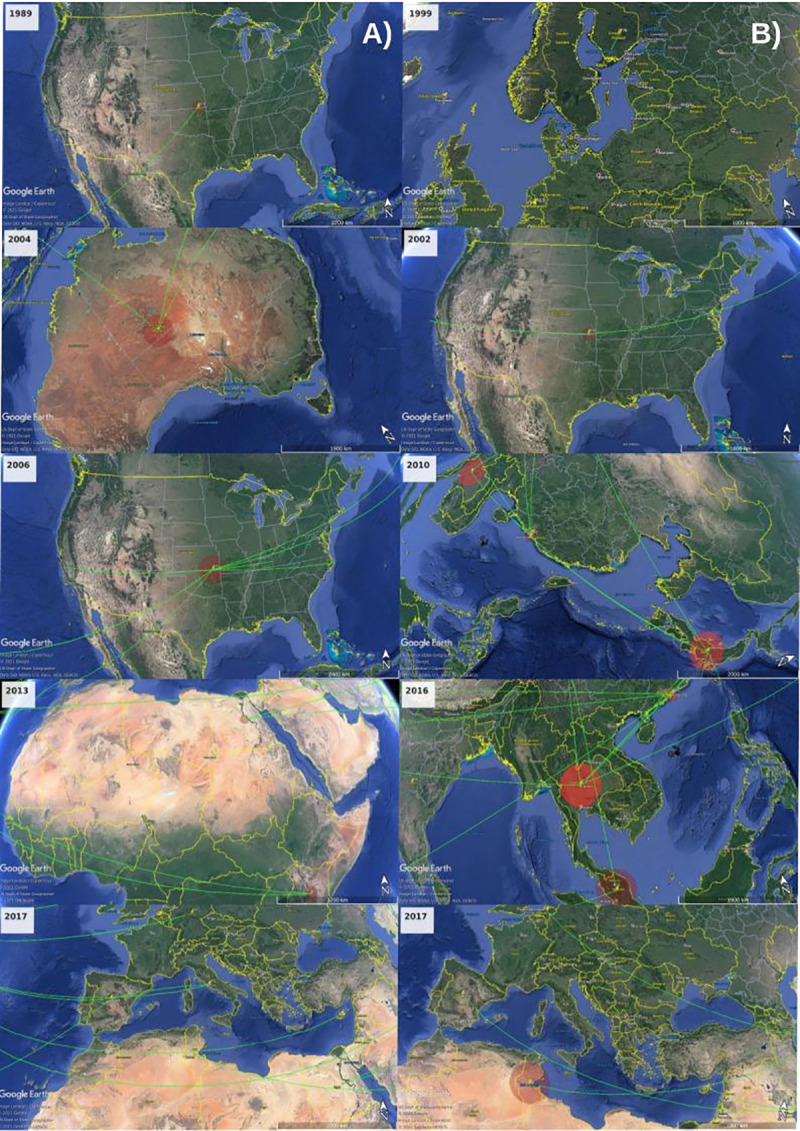
Temporal dynamics of RV-A101 (Panel. A) and RV-C45 (Panel. B) and spatial dispersal in the world. Google Earth (https://earth.google.com). The figure is similar but not identical to the original image and is therefore for illustrative purposes only.

Phylogeography generally describes the geographical distribution of lineages and has been used to reconstruct the geospatial dynamics of disease spread and the diffusion process of RV-A101 and RV-C45 in Tunisia and in the world by mapping the spatial estimates annotated in the MCC trees on Google Earth Pro version 7.3 (http://www.google.com/earth/download/ge/). This mapping allows visualizing the virus’s geographic spread process over time. The links between different geographic regions represent branches in the MCC tree on which virus migration occurs and circle areas reflect the number of branches maintaining a particular location at that time point. The panels in Fig 3 show the temporal dynamics of RV-A101(Fig 3A) and RV-C45 (Fig 3B) spatial dispersal processes in the world.

Our results suggest that the most ancient RV-A101 virus dated around 1981 in the world. Based on the investigated RV-A101 sequences, the phylodynamic and phylogeographic analysis revealed that the virus started its migration from the USA around 1981 to Australia in 1988, Asia (the Philippines and South Korea) starting from 2003, European countries including United Kingdom, Germany, Spain, and Italy from 2005 to 2009 than East Africa around 2010. The USA would have been the origin of the spread to other countries worldwide. The results showed that RV-A101 isolates were introduced to the USA several times.

The virus spread in South Korea and reached India, Singapore, and China in 2011. It migrated into Colombia through Spain around 2013. Concerning North Africa, a migration of the virus from China to Egypt would have occurred in 2011 and then to Taiwan in 2012. From the USA, the virus would have reached Malaysia in 2011 where it spreads from 2011 to 2015, then migrated to Tunisia around 2016–2017.

For the RV-C45, the virus seems to have migrated from Finland around 1998 to the USA in 2001 where it started to spread then continued its migration to reach Japan in 2004 and from there to Thailand, around 2006, where it spread until 2007. During this period, the virus migrated from Japan to Argentina and Egypt around 2012 and from Thailand to China around 2010. In 2011, the virus migrated again from Thailand to Japan and reached Cyprus in 2014. Meanwhile, in 2013 RV-C45 migrated from the USA to Mongolia, Japan, and Thailand. RV-C45 then migrates from Mongolia to reach Tunisia in 2016 and Malaysia around 2015. It spreads in Tunisia until 2017 then migrates into India in 2018.

### Phylogenetic analysis of other circulating RV-A

**Fig 4:**
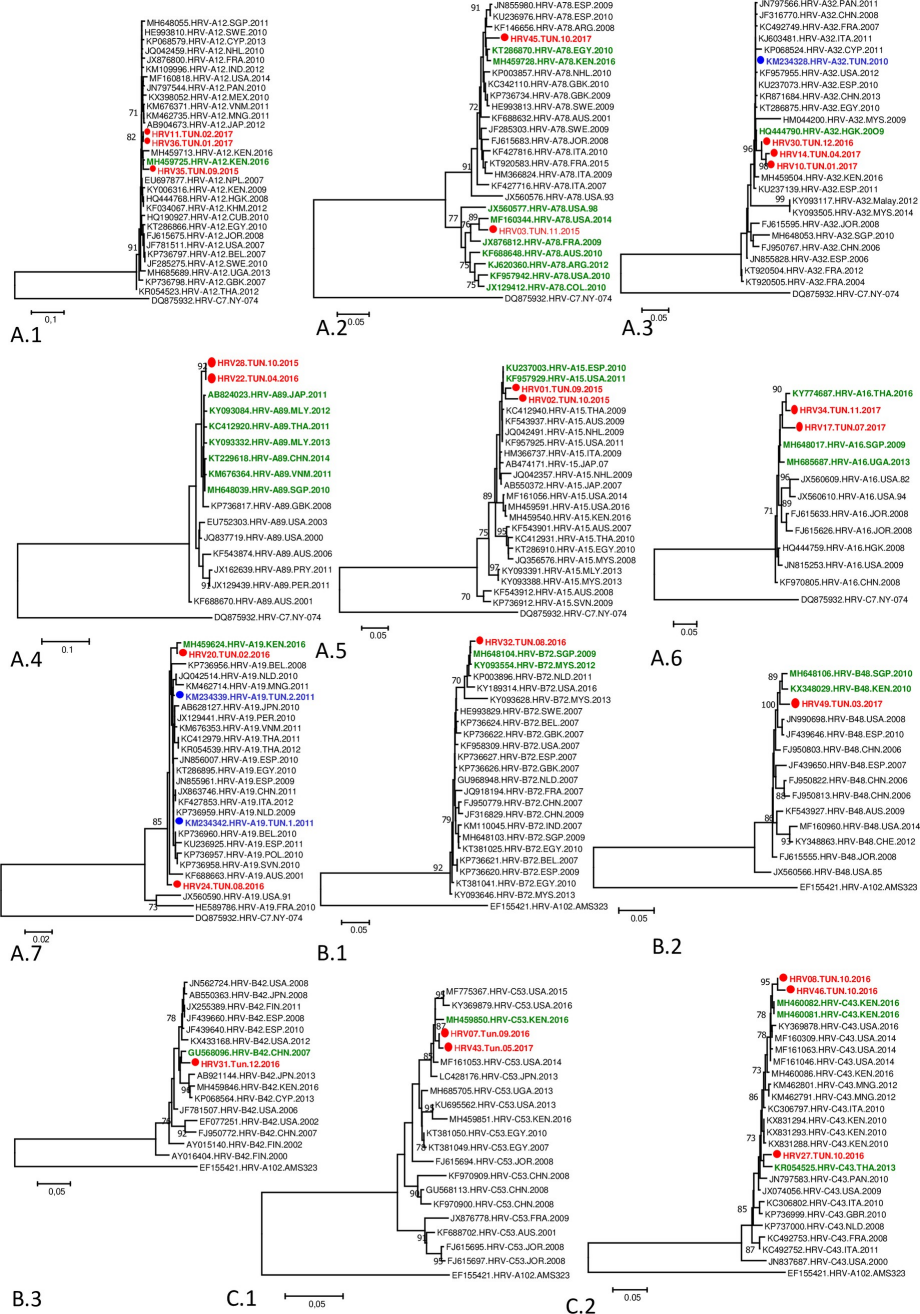
Molecular typing of Tunisian RV strains obtained during the study period (2015–2017). **A. Molecular typing of RV-A strains**: A1. RV-A12, A2. RV-A78, A3. RV-A32, A4. RV-A89, A5. RV-A15, A6. RV-A16, A7. RV-A19; **B. Molecular typing of RV-B strains**: B1. RV-B72, B2. RV-B48, B3. RV-B42; **C. Molecular typing of RV-C strains**: C1. RV-C53, C2, RV-C43. The evolutionary history was inferred using the Neighbor joining method. The percentage of replicate trees in which the associated taxa clustered together in the bootstrap test (1000 replicates) is shown next to the branches. Evolutionary analyses were conducted in MEGA6. Investigated Tunisian sequences are indicated in bold and Red; The most related sequences to investigated ones are indicated in bold and Green; Previously available Tunisian are indicated in bold in Blue.

In the aim to analyze genetic variability and identify the epidemiological origin of RV strains circulating in Tunisia, during the study period, we assessed phylogenetic analyses of strains circulating during at least 5 months (RV-A12, RV-A78, RV-A32, RV-A89, RV-A15, RV-A16, RV-A19, RV-A18) (Fig 4A).

Three strains of RV-A12 were detected in 2015 and 2017. The two strains circulating in 2017 were identical and slightly different from the one that circulated in 2015 (96.1% identity). With other Kenyan strains, they seem to constitute an independent clade confirmed by a high bootstrap value (82%). The most related strain was a Kenyan strain (MH459725) detected in 2016 (98.1–98.8% identity) (Fig 4A.1).

Two RV-A78 Tunisian strains were detected in 2015 and 2017, the are genetically quite different (90.2% identity) and belong to two different clades confirmed by high bootstrap values (77% and 91%): the one detected in 2015 was highly related to strains that originated from the Americas and especially to a strain isolated in 2014 (MF160344) with 96.2% identity rate. The strain detected in 2017 clustered with African strains detected in Egypt (97.3% identity rate) and Kenya (97.1% identity rate) (Fig 4A.2).

Regarding RV-A32, three Tunisian strains were detected in 2016 and 2017. The two strains from 2017 shared 99.6% nucleotide identity; and had 99.3%, 98.9% identity rates with the strain from 2016. These strains were deeply related to a strain from Hong Kong (HQ444790) with identity rates ranging from 98% to 99.1%. They constitute an independent clade supported by a high bootstrap value (96%). Another Tunisian strain (RV-A32) obtained in 2010, was previously published in GenBank. However, it is slightly different from the 2016 and 2017 Tunisian sequences (Identity rates ranging from 97.2% to 98%) (Fig 4A.3).

For RV-A89, two Tunisian strains were detected in 2015 and 2016; they are identical and closely related to Pacific Asiatic strains that circulated between 2010 and 2014 (98–98.4% identity rate) (Fig 4A.4).

Two strains of RV-A15 were detected, in Tunisia, in 2015 and 2016. Both are closely related to each other (99% identity rate) and with strains from the USA (KU237003) and Spain (KF957929) with 98.3% and 99.3% identity rates, respectively (Fig 4A.5).

The two RV-A16 Tunisian strains here investigated were slightly different from each other (96% identity rate). and clustered with pacific Asian strains. The HRV34.TUN.11.2017 strain was closely related to a strain obtained in Thailand in 2014 (98.6% identity) while the HRV17.TUN.07.2017 strain was slightly different and presented approximately the same identity rates with all strains included in this clade (95.8–96% identity rates) (Fig 4A.6).

For RV-A19, the phylogenetic tree grouped all strains into two clusters supported by high bootstrap values (73 and 85%). The newly investigated Tunisian strains are different from each other. Indeed, the strain HRV24.TUN.08.2016 seems to be genetically independent but at the same time, it shares an identity rate of 98% with a Peruvian strain (JX129441) taken in 2010. However, the strain HRV20.TUN.02.2016 is related to a Kenyan strain (MH459624) with an identity rate of 99.2%. Other Tunisian strains, obtained in 2011, were available in GenBank. They seem to belong to different clades. The first strain (KM234342) is identical to an Egyptian (KT286895), Spanish (JN855961), and Netherlander strains (KP736959). The second Tunisian strain (KM234342) was related to another Netherlander (JQ042514) and Mongolian (462714) strains (99.1 and 98.9% identity rates respectively) (Fig 4A.7).

### Phylogenetic analysis of RV-B

All three detected RV-B viruses were investigated (Fig 4B). Phylogenetic analysis of RV-B72 demonstrates that the Tunisian strain is strongly related to strains from Singapore (MH648104) obtained in 2009 and Malaysia (KY09355) in 2012, with an identity rate of 99.1 and 98.9%, respectively (Fig 4B.1). Regarding RV-B48, the Tunisian strain is related to Kenyan (KX348029) and Singaporean (MH648106) strains, obtained in 2010with an identity rate of 97.4% (Fig 4B.2). Also, for RV-B42, the Tunisian strain was deeply related to a Chinese (GU568096) strain obtained in 2007 with a 97.3% identity rate (Fig 4B.3).

### Phylogenetic analysis of other circulating RV-C

Regarding RV-C, we are interested in the most circulating types, either RV-C45: RV-C53; RV-C43 (Fig 4C).

Two Tunisian strains of RV-C53 were circulating in Tunisia in 2016 and 2017. Both strains were closely related to each other (99.3% identity rate) and with a strain from Kenya (MH459850) (97.8 and 98.6% identity rates respectively) (Fig 4C.1).

Regarding RV-C43, three Tunisian strains were detected during the study period. Among them, HRV08.Tun.10.2016 and HRV46.Tun.10.2016 were closely linked to each other (99.1% identity rate) and are related to the Kenyan strains (MH460082, MH460081) with 97.7 and 98.6% identity rates respectively. In the other hand, the HRV27.Tun.10.2016 strain was closely related to a strain obtained in Thailand (KR054525) with 98% identity rate (Fig 4C.2).

## Discussion

RVs represent one of the leading causes of hospitalization and mortality in neonates and young children. Thus, their molecular characterization is of major interest to understand their epidemiology and genetic evolution. Previous studies focused mainly on the prevalence and type diversity of circulating RVs, with few data on the phylogeny and phylogeography of the different types [12–14]. In-depth molecular studies of circulating strains can help understanding the genetic diversity within each type, identifying endemic and persistent clades in a region and evaluating the contribution of virus importation and exchange from a region to another in the virus spread. To the best of our knowledge, this is the first study describing the phylogeny and phylogeography of the RV types circulating in a region.

In this study, phylogeographic analyses were conducted on the most frequently detected RV types in Northern Tunisia (RV-A101 and RV-C45), from September 2015 to November 2017 [16]. Both types belong to the most frequently circulating RV species in the world (A and C); they were detected in Europe, the USA, Asia, and Africa [11]. Tunisian strains were closely related to each other and to other strains from Asia suggesting importation events from Asiatic countries followed by the dissemination of different strains from the same clade during 11 and 4 months, respectively. After January and June 2017, and during the study period, the circulation of both types seems interrupted and, likely, replaced by other RV types. The global topology of the phylogenetic trees showed at least four potential monophyletic groups of RV-A101 and RV-C45 that circulated in the world. However, the evolution and spread of virus clusters do not respect any geographic distribution; they seem disseminated throughout the world without any geographic restriction. This finding may indicate a regular exchange of strains from one region to another and supports the hypothesis that circulation of RV-A101 and RV-C45 occurs through regular introductions of viruses from different clades rather than a local persistence of the same clade from a season to another. Unfortunately, a very restricted number of RV sequences from several seasonal epidemics in Tunisia are available. Nevertheless, considering the available RV sequences, our phylogenic analysis of other RV-A and RV-C types displayed a remarkable homogeneity among strains obtained during the same seasonal period; while comparing to strains from 2011, they seem different and related to strains from distinct geographic origins. The same result was also obtained for RV-A78 and RV-A12 sequences where strains obtained in 2015 were related to strains from different geographic origins compared to strains obtained in the following epidemic period in 2017. This suggests that, in general, the genetic diversity of RV types results from regular introductions of new viruses rather than a continuous local evolution of specific genotypes. Long-term surveillance of RV circulating types during multiple seasonal periods may further support this finding Also, investigation of another genetic region, such as the VP1 region or full genomic RV sequences may support our hypothesis. In our study, given the limited number of samples obtained from young children and the multiple bacterial and virologic investigation conducted for diagnosis, the residual amounts of samples were only sufficient for sequencing the VP2-VP4 region. Other studies concerning other respiratory viruses such as the influenzae virus revealed that the genetic diversity is also a result of frequent introductions of genetically divergent viruses from different clades during seasonal epidemics [25–27]. For instance, genetic analysis of the H3N2 virus over 15 years in China did not demonstrate any continuous virus clade persistence over seasonal epidemics [28].

On the other hand, phylogeographic analyses highlights the significant spread of both virus types from one country to another. The present work suggests that RV-A101 and C45 progenitor viruses rise approximately in the eighteenth and nineteenth, respectively. Since the early 2000s, they knew a big spread all over the world and were detected in The United States of America, Australia, Asian countries, European countries, and some African countries, despite the restricted number of available sequences from some regions. The level of international viral traffic was high and rapid which suggests travel-related dissemination of RV-A101 and C45. Given the mild clinical presentation of RV infection in adults, travelers may significantly contribute to the spread of RVs, globally. Furthermore, phylogenetic analysis of other detected RVs circulating in Tunisia revealed strong epidemiological links with strains from the USA, Asian and African countries. During our study period, different virus types from different geographic origins co-circulated simultaneously, with extinction and replacement of some strains by other strains from the same type or other types but in general from different geographic origins.

## Conclusions

In this study, the genetic diversity of the most frequently circulating RVs was investigated, during two successive SARI epidemic periods in Northern Tunisia, using phylogeny and phytogeography. It highlights the high genetic homology among viruses from the same type during a seasonal epidemic period with lower homology rates with strains from another period. The study suggests high international spread of RV-A101 and RV-C45. Thus, circulation of RV-A101 and RV-C45 seems to result from regular introductions of viruses from different clades followed by their local dissemination, rather than a local persistence of viruses from the same clade along seasons. International traffic may play a pivotal role in the spread of RV-A101, RV-C45, and other RV types. Long-term surveillance of RV circulating types during multiple seasonal periods may further support these findings.

## Supporting information

S1 Table Description of RV-A101 sequences included in this study. (DOCX)

S2 Table Description of RV-C45 sequences included in this study. (DOCX)

S1 Fig Smart selection tool output of RV-A101 and RV-C45. (JPG)
