# Supplementary material for: Phylogeography and phylogeny of Rhinoviruses collected from Severe Acute Respiratory Infection (SARI) cases over successive epidemic periods in Tunisia
Source: PLoS One. 2021 Nov 22;16(11):e0259859. doi: 10.1371/journal.pone.0259859 (PMC8608298; doi:10.1371/journal.pone.0259859)
Supplement: S1 Table — (DOCX) [file pone.0259859.s001.docx]

**S1 Table: Description of HRV-A101 sequences included in this study**

| **Accession Number** | **Year of Isolation** | **Country*** |
| --- | --- | --- |
| KF688694 | 2001 | AUS |
| KF688693 |  |  |
| KF688665 |  |  |
| KF688715 |  |  |
| KF688636 |  |  |
| KF688622 |  |  |
| KF688695 |  |  |
| KF688655 |  |  |
| KF688633 |  |  |
| KF688660 |  |  |
| KF688641 |  |  |
| KF688610 |  |  |
| GU568108 | 2007 | CHN |
| GU568107 |  |  |
| EU822866 |  |  |
| KF970812 | 2008 |  |
| KF970798 |  |  |
| KF498952 | 2012 |  |
| KF498947 |  |  |
| KR871692 | 2013 |  |
| KP754484 | 2014 |  |
| KP754500 |  |  |
| JX129413 | 2011 | COL |
| KP068585 | 2013 | CYP |
| EU081786 | 2006 | DEU |
| EU081780 |  |  |
| EU081785 |  |  |
| KT286867 | 2010 | EGY |
| JN855831 | 2006 | ESP |
| EU697826 | 2007 |  |
| KU237024 | 2010 |  |
| GQ476595 | 2006 | GBR |
| GQ476608 |  |  |
| GQ476605 |  |  |
| GQ476605 |  |  |
| HQ444778 | 2008 | HKG |
| HQ444757 |  |  |
| HQ444760 |  |  |
| HQ444794 | 2009 |  |
| KM109978 | 2009 | IND |
| HM366808 | 2008 | ITA |
| HM366809 |  |  |
| FJ615693 | 2008 | JOR |
| FJ615692 |  |  |
| FJ615691 |  |  |
| FJ615690 |  |  |
| FJ615688 |  |  |
| FJ615687 |  |  |
| FJ615689 |  |  |
| KY006293 | 2008 | KEN |
| KX831164 | 2010 |  |
| KX831163 |  |  |
| KX831162 |  |  |
| KX831161 |  |  |
| KX831160 |  |  |
| MH459638 | 2015 |  |
| MH459643 | 2016 |  |
| MH459642 |  |  |
| MH459641 |  |  |
| MH459639 |  |  |
| MH459640 |  |  |
| FJ869938 | 2006 | KOR |
| FJ869949 |  |  |
| FJ869945 |  |  |
| KY093237 | 2012 | MYS |
| KY093207 |  |  |
| KY093463 | 2013 |  |
| KY093305 |  |  |
| KY093467 | 2014 |  |
| KY093469 |  |  |
| AB683942 | 2001 | PHL |
| MH648015 | 2008 | SGP |
| KR054529 | 2011 | THA |
| KY460514 | 2010 | TWN |
| JX560586 | 1986 | USA |
| JQ245965 | 1999 |  |
| JX560585 | 2000 |  |
| GQ415051 |  |  |
| JX560584 | 2001 |  |
| EF077258 | 2004 |  |
| EF077259 |  |  |
| GQ415052 | 2008 |  |
| MF160952 | 2014 |  |
| MF161051 |  |  |
| MF161013 |  |  |
| MF160961 |  |  |
| MF160934 |  |  |
| MF161003 | 2014 | USA |
| MF160974 |  |  |
| MF160964 |  |  |
| MF160940 |  |  |
| MF160971 |  |  |
| KY369892 | 2016 |  |
| KY369889 |  |  |
| KY369897 |  |  |
| KY189315 |  |  |
| KY369891 |  |  |

(*) : Alpha-3 country code
