# Supplementary material for: Phylogeography and phylogeny of Rhinoviruses collected from Severe Acute Respiratory Infection (SARI) cases over successive epidemic periods in Tunisia
Source: PLoS One. 2021 Nov 22;16(11):e0259859. doi: 10.1371/journal.pone.0259859 (PMC8608298; doi:10.1371/journal.pone.0259859)
Supplement: S2 Table — (DOCX) [file pone.0259859.s002.docx]

**S2 Table**: **Description of HRV-C45 sequences included in this study**

| **Accession number** | **Year of isolation** | **Country** |
| --- | --- | --- |
| KF146694 | 2009 | ARG |
| KF970899 | 2008 | CHN |
| KF970882 |  |  |
| KP068563 | 2012 | CYP |
| KT381086 | 2010 | EGY |
| JF897694 | 2008 | ESP |
| JF897753 | 2009 |  |
| EU590064 | 1996 | FIN |
| HQ444845 | 2008 | HKG |
| HQ444913 | 2009 |  |
| HQ444911 |  |  |
| KY379152 | 2016 | IND |
| AB550398 | 2008 | JPN |
| AB548907 | 2009 |  |
| AB550401 |  |  |
| AB831599 | 2012 |  |
| KY006361 | 2008 | KEN |
| KY006362 | 2009 |  |
| KM462795 | 2012 | MNG |
| KY093961 | 2013 | MYS |
| KY094024 | 2014 |  |
| JQ042320 | 2009 | NLD |
| KR922047 | 2010 | THA |
| KC412959 | 2011 |  |
| KR922048 |  |  |
| KR054531 | 2012 |  |
| KY624849 | 2013 | UGA |
| JN837686 | 2000 | USA |
| MF160342 | 2014  2014 |  |
| MF160998 |  |  |

(*) : Alpha-3 country code
