# Supplementary figures and images for: Phylogeography and phylogeny of Rhinoviruses collected from Severe Acute Respiratory Infection (SARI) cases over successive epidemic periods in Tunisia
Source: PLoS One. 2021 Nov 22;16(11):e0259859. doi: 10.1371/journal.pone.0259859 (PMC8608298; doi:10.1371/journal.pone.0259859)

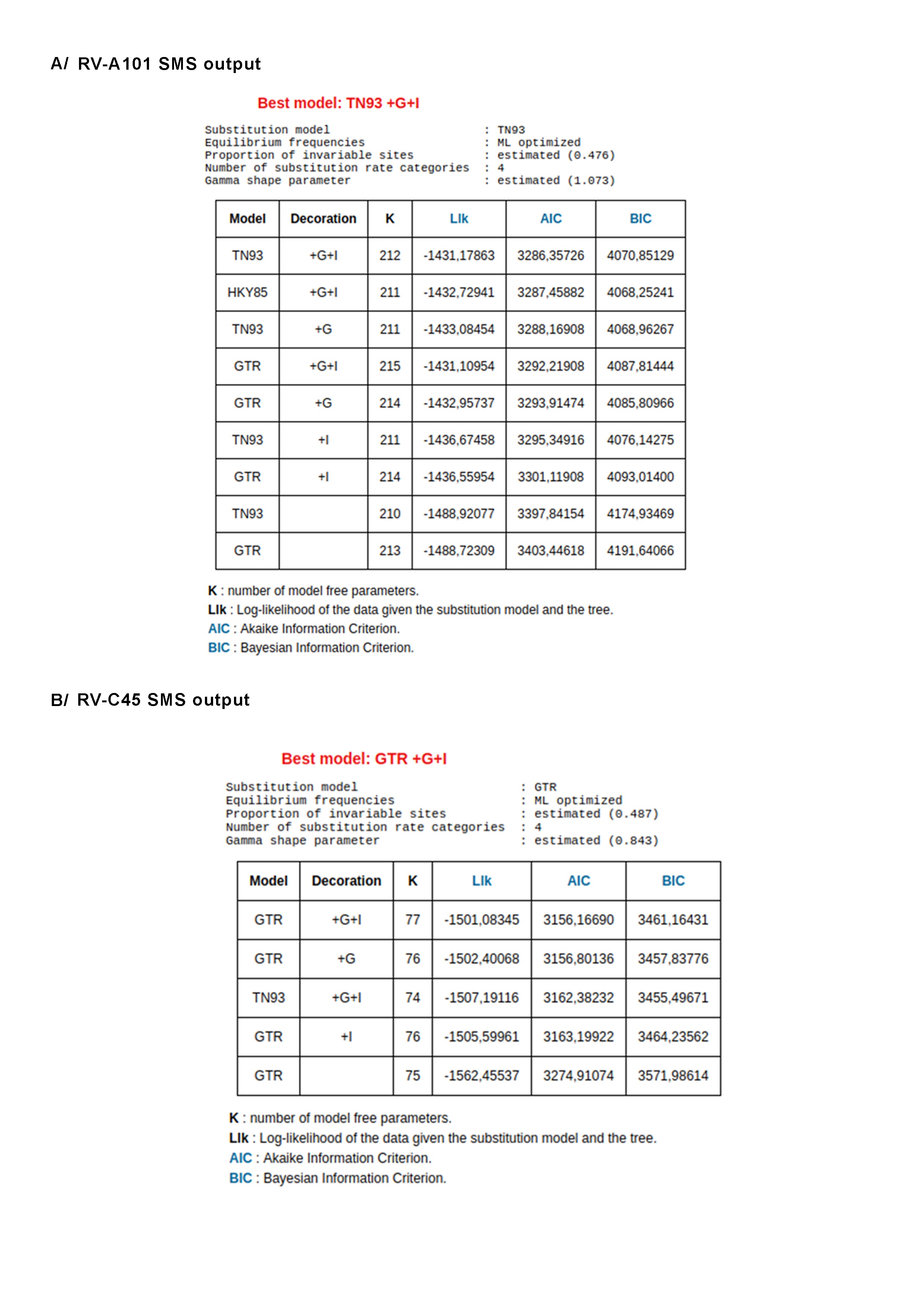

Supplement: S1 Fig — (JPG) [file pone.0259859.s003.jpg]
